# A molecular clone of Chronic Bee Paralysis Virus (CBPV) causes mortality in honey bee pupae (*Apis mellifera*)

**DOI:** 10.1038/s41598-019-52822-1

**Published:** 2019-11-07

**Authors:** Kerstin Seitz, Katharina Buczolich, Alžbeta Dikunová, Pavel Plevka, Karen Power, Till Rümenapf, Benjamin Lamp

**Affiliations:** 1 Institute of Virology, Department of Pathobiology, University of Veterinary Medicine Vienna, Veterinaerplatz 1, 1210 Vienna, Austria; 2 Structural Virology Unit, Central European Institute of Technology, Masaryk University, Kamenice 753/5, 62500 Brno, Czech Republic; 3 Department of Veterinary Medicine and Animal Production, University of Naples “Federico II”, Via Delpino, 1, 80137 Naples, Italy; 4 Present Address: Institute of Virology, Faculty of Veterinary Medicine, Justus-Liebig-University, Schubertstrasse 81, 35392 Giessen, Germany

**Keywords:** Viral pathogenesis, Viral transmission

## Abstract

Among the many diseases compromising the well-being of the honey bee (*Apis mellifera*) the chronic paralysis syndrome of adult honey bees is one of the best described. The causative agent, chronic bee paralysis virus (CBPV), is a positive sense, single-stranded RNA virus with a segmented genome. Segment 1 encodes three putative open reading frames (ORFs), including the RNA-dependent RNA polymerase and other non-structural protein coding regions. Segment 2 encodes four putative ORFs, which contain the genes of supposed structural proteins. In this study, we established a reverse genetic system for CBPV by molecular cloning of DNA copies of both genome segments. CBPV rescue was studied in imago and honey bee pupae infection models. Virus replication and progeny virus production was only initiated when capped RNAs of both genome segments were injected in honey bees. As injection of these clonal RNAs caused clinical symptoms similar to wild-type CBPV infection, we conclude that the novel molecular clone fulfilled Koch’s postulates. Our virus clone will enable in-depth analysis of CBPV pathogenesis and help to increase knowledge about this important honey bee disease.

## Introduction

The chronic bee paralysis virus (CBPV) is the causative agent of severe, usually fatal, paralysis in adult honey bees^1^. CBPV has not been assigned to any group or class of viruses by the International Committee on Taxonomy of Viruses (ICTV) because it has a unique genome organization and virion morphology^2^. However, analyses of the nucleic acid sequence showed similarities between the RNA-dependent RNA polymerase (RdRp) of CBPV and the RdRps of Tombusviruses, Nodaviruses, Lake Sinai virus 1 and 2 as well as the Anopheline associated C virus (AACV)^3–6^.

CBPV was one of the first viruses in honey bees to be described and isolated as it causes characteristic signs of disease in adult honey bees^7,8^. Clinical signs of CBPV infection include two major syndromes; most affected bees show bloated abdomens, dislocated wings, and flight inability resulting in an accumulation of abnormally trembling and paralyzed bees in front of the hive^9^. Some CBPV affected individuals develop a hairless, shiny, black appearance and are usually aggressively driven out of the hive by guard bees^10^. CBPV symptoms are often misinterpreted and overlooked in the fall as the beekeeper considers the fights at the hive entrance as robber bee attacks^11^. It is believed that the natural transmission of CBPV within the colony is mediated by close contact between infected and healthy bees and by the oral uptake of infectious particles from faeces of diseased animals^12,13^. CBPV appears to have a wider host range as it has been found in two different ant species (*Camponotus vagus* and *Formica rufa*). Detection of CBPV nucleic acid in the parasitic Varroa mite (*Varroa destructor*) may indicate another possible route of virus transmission^14^. However, the low CBPV RNA copy numbers measured in ants and mites make it questionable whether these species are actually true hosts of CBPV or just passive carriers of infectious material. Honey bees can be infected with CBPV by topical or oral application and via injection under laboratory conditions^7^. Very high CBPV genome loads exceeding 1 × 10^13^ genome equivalents (GE)/bee occur in symptomatic CBPV-infected adult worker bees^15^. However, the virus is able to infect honey bees at all developmental stages, with previous studies measuring low genome loads of about 2 × 10^3^ GE/bee in honey bee pupae and larvae in natural infections^12^.

In contrast to the picornavirus-like virions of other bee viruses^16,17^, CBPV particles have a pleomorphic, oval appearance and a variable size, with a width of 20–30 nm and a length of up to 60 nm^18^. The genome consists of two small single stranded RNA molecules (bipartite genome) of positive polarity, termed RNA1 (3.6 kb) and RNA2 (2.3 kb)^6,19,20^. While RNA1 contains the genes encoding enzymes of the viral replicase, RNA2 harbours the genes whose products are probably glycosylated structural proteins^6,21,22^. Recent studies showed that the injection of the two small CBPV RNA segments is sufficient to initiate viral replication in honey bees^20^. It has been suggested that the genome segments of CBPV carry a 5′-cap structure, because ORF1 of RNA1 contains domains homologous to the methyltransferase guanylyltransferase gene of alphaviruses which is involved in cap formation^22^. The 5′-cap structure consists of a guanine nucleotide connected to the 5′ end of mRNA via a 5′-5′ triphosphate linkage. The guanine is methylated by a methyltransferase at position 7, hence the name 7-methylguanylate cap (m^7^G-Cap). 5′-cap structures are essential for the efficient gene expression of mRNA molecules and protect RNA molecules from degradation by cellular exonucleases^23^. In this study, we have established a reverse genetics system for CBPV to test whether 5′-cap structures are indeed essential for viral replication.

## Material and Methods

### Honey bees

Adult honey bees (*Apis mellifera carnica*) and bee pupae for the experiments were collected from the apiary of the University of Veterinary Medicine Vienna with the permission of the institution. For each experiment, worker bee pupae of the same age were taken from a single comb, which had been freshly introduced in the colony and subsequently marked after egg deposition to calculate the age of pupae. We extracted 13 to 15 days old pupae from capped brood cells using spring steel tweezers, transferred the pupae in a well of a 24-well tissue culture plate, and placed them at 35 °C in a humidified incubator. In order to collect freshly emerged honey bees, we harvested capped combs made free of adult nurse bees at day 20 post egg deposition and incubated the combs in plastic bags under laboratory conditions. Freshly emerged adult worker bees (day 21 of development) were collected the next day and caged in groups of 25 individuals.

### Infection of honey bees

On the day after extraction, healthy looking pupae showing no signs of injury from the extraction process were either transfected with synthetic RNAs from our recombinant molecular clones (rCBPV AUT17-CBP1 and rCBPV AUT17-CBP2), infected with wild type virus (wtCBPV) preparations, or mock infected with phosphate buffered saline (PBS). After 3 to 7 days of incubation, pupae were snap-frozen at −80 °C in Eppendorf vials and further processed as described in a previous study^24^. Adult bees were maintained in an incubator at 35 °C for 10 days for the topical infection experiment and fed *ad libitum* with invert sugar syrup (Apiinvert, Südzucker, Mannheim, Deutschland). During the experiment, deceased bees were collected and stored at −80 °C for further analysis. At the end of the experiment, the remaining bees were snap-frozen at −80 °C in Eppendorf vials and further processed. Individual honey bees were homogenized in 500 µl sterile distilled DEPC water using 5 mm stainless steel beads (Qiagen, Hilden, Germany) in a TissueLyser II (Qiagen) for 3 min at 30 Hz.

### CBPV field sample

A beekeeper from Lower Austria reported typical clinical signs of CBPV in his apiary in the summer of 2017. In particular, paralyzed bees were observed at the entrance of a hive, together with several hairless bees being expelled from the hive and attacked by guard bees (Additional Video Files 1 and 2). A sample of 50 dead adult honeybees from the bottom board of the affected colony was collected for diagnostic purposes. The bee sample was evaluated macroscopically and ten bees were selected for further analysis, based on their dark discoloration typical for CBPV infection. The bees were homogenized in 5 ml sterile distilled DEPC water and total RNA was purified from 140 µl of the clarified and sterile filtrated lysate. Diagnostic laboratory tests confirmed the presence of CBPV nucleic acids in the diagnostic sample. Additionally, the sample was tested negative in well-established diagnostic RT-PCR assays for Deformed wing virus (DWV)^24^, Sacbrood virus (SBV)^25^, and Acute bee paralysis virus (ABPV)^26^. With the consent of the beekeeper, we used the sample to isolate the virus (wtCBPV- Aut17) and to study its pathogenicity under controlled laboratory conditions.

### Virus stock preparation and purification

Bee pupae and adult bees were transferred to individual Eppendorf tubes and euthanized by freezing at −80 °C. For virus stock preparation and RNA extraction, the honey bees were homogenized in 500 µl sterile distilled DEPC water using 5 mm stainless steel beads (Qiagen, Hilden, Germany) in a TissueLyser II (Qiagen) for 3 min at 30 Hz. The resulting bee lysate was cleared by centrifugation (13.000 rpm for 1 min) and filtered through a 0.45 μm nylon filter (Acrodisc, Sigma-Aldrich, St. Louis, Missouri, USA). The crude bee lysates were aliquoted and RNA from one aliquot (140 µl) per sample was extracted to measure the CBPV load by quantitative RT-PCR (RT-qPCR). No virus purification was used, such as particle preparation by gradient centrifugation or chromatography, in order to not impair the infectivity of the virions. The virus stocks were tested for the presence of other common bee viruses by conventional RT-PCR (SBV, ABPV) or RT-qPCR (DWV), respectively. The sequences of oligonucleotides used in this study are listed in Table 1.

**Table 1:** Oligonucleotides used in this study.

Name	Reference	RNA	Sequence (5′-NNN-3′)	Strand orientation
CPV1		1	CGCAAGTACGCCTTGATAAAGAAC	forw. nt 2391
CPV2		1	ACTACTAGAAACTCGTCGCTTCG	rev. nt 2469
CPV3		1	FAM-TCAAGAACGAGACCACCGCCAAGTTC-TAMRA	forw. nt 2419
CPV4		1	GGCCACGCGTCGACTAGTACCTAAATGTGGTGAAGTGCG	rev. nt 3588
CPV5		1	GTTGGTAACTTGGACGATC	rev. nt 370
CPV6		1	ATTGCGGCTGCGATCGGATG	rev. nt 176
CPV7		1	GGCCACGCGTCGACTAGTACGGGCTACCTTACCAGTGC	rev. nt 3662
CPV8		1	ATTTAGGTGACACTATAGGTAAACTTTAGGACTAAGATGAATC	forw. nt 1
CPV9		1	TAGCAGCGGCCGCAACGGGGCTACCTTACCAGTGCCTT	rev. nt 3679
CPV10		1	ATCGGTGCTCTGACATATGC	forw. nt 3228
CPV11		1	ACCACATTTAGAGCCAC	forw. nt 3580
CPV12		1	CTCGTAGACTGCGTACACGAC	3′-RACE
CPV13		1	PHO-GTCGTGTACGCAGTCTACGAGGTACTAGTCGACGCGTGGCC-AmC3	3′-RACE
CPV14		1	GGCCACGCGTCGACTAGTACTTTTTTTTTTTTTTTTT	5′-RACE
CPV15		1	GGCCACGCGTCGACTAGTAC	5′-RACE
CPV16		1	ACGGCACTGAATATCAGC	forw. nt 906
CPV17		1	ATCGAAACCAGAGTGAT	forw. nt 1710
CPV18		1	ATCGGTGCTCTGACATATGC	forw. nt 3228
CPV19		1	GAGACCACCGCCAAGTTCGTG	forw. nt 2427
CPV20		2	GGCCACGCGTCGACTAGTACAACCATGATAGGGCGTATGACGAC	rev. nt 2282
CPV21		2	AGCGTGAACGGGCCATCGCAC	rev. nt 299
CPV22		2	ACCTGAGATAACGTAGGCC	rev. nt 163
CPV23		2	GGCCACGCGTCGACTAGTACGGGAACCATGATAGGGCGTA	rev. nt 2308
CPV24		2	TAGCAGCGGCCGCAACGGGGAACCATGATAGGGCGTATG	rev. nt 2308
CPV25		2	ATTTAGGTGACACTATAGGTAAACCTTAGGCTTTCATC	forw. nt 1
CPV26		2	CATTCTCCTCAGCGTCTTTGCTCTCGGCGTTTATG	forw. nt 768
CPV27		2	TCCTCGATGGTTCGGAGTTGGAG	rev. nt 742
CPV28		2	ATTCCTGGCTCAAGAAAGC	forw. nt 1807
CPV29		2	CAATAACGCGGTCAACACCAC	forw. nt 2090
CPV30		2	ACTCTCAATGAGAAGTACCGTGTC	forw. nt 1911
CPV31		2	GATCTTGATGTCAGCTGATACCAAG	rev. nt 1625
CPV32		2	TCTAACATCCACCGAGACGTTAAC	forw. nt 1650
CPV33		2	ACATACCATTATAAGCACCAATC	forw. nt 1319
CPV34		2	ATCAAGGTGTCAACGACTGAC	rev. nt 1844
CPV35		2	GATCTTGATGTCAGCTGATACCAAG	rev. nt 1625
CPV36		2	TCTAACATCCACCGAGACGTTAAC	forw. nt 1650
fDWV qRT	Berényi *et al*., 2007		ATTGTGCCAGATTGGACTAC	forw.
rDWV qRT	Berényi *et al*., 2007		AGATGCAATGGANGATACAG	rev.
fSBV RT	Grabensteiner *et al*., 2001		ACCAACCGATTCCTCAGTAG	forw.
rSBV RT	Grabensteiner *et al*., 2001		CGTGTAATTATTGTATTNTCCTC	rev.
fABPV RT	Bakonyi *et al*. 2002		GTGCTATCTTGGAATACTAC	forw.
rABPV RT	Bakonyi *et al*. 2002		AAGGTTTAGGTTCTACTACT	rev.

Po**s**ition refers to MK637522 for RNA1 and MK637523 for RNA2 in GenBank. Adapter primer and vector sequences, which are not related to CBPV sequences are underlined.

### RNA extraction and RT-qPCR

Total RNA was extracted from bee lysates of rCBPV, wtCBPV, and negative controls using the QIAmp Viral RNA Mini Kit together with the QIAcube (Qiagen) according to the manufacturer’s instructions. RNA was eluted in 60 µl RNase free elution buffer and stored at −80 °C for subsequent analyses. A real-time one-step TaqMan probe-based RT-qPCR assay was performed for CBPV quantification using previously published oligonucleotides within the putative RdRp region of CBPV RNA1^12^. The assay was run on an Applied Biosystems 7500 real-time PCR system and evaluated using the 7500 System SDS Software (Applied Biosystems, Foster City, USA). A 10-fold serial dilution of a DNA plasmid containing the CBPV target sequence served as a cDNA standard allowing absolute quantification of genome equivalents. The genome equivalents from 1 µl extracted RNA were projected to the genome load per bee using a multiplication factor of 214,29. The samples were analyzed in duplicate runs to ensure proper measurements together with the necessary extraction and “no template” controls.

### Nucleotide sequence determination

The nucleotide sequences of RNA1 (AUT17-CBP1; GenBank MK637522) and RNA2 (AUT17-CBP2; GenBank MK637523) of the CBPV strain were determined using subgenomic RT-PCR products and Sanger sequencing (Eurofins Genomics, Ebersberg, Germany) to have all the information necessary for molecular cloning. After generating a first raw consensus sequence, selected PCR products covering the entire genome were inserted into a T-vector backbone (pGEM T-easy, Promega, Fitchburg, USA). We repeated the sequencing using the plasmid derived DNA to ensure high quality results. Oligonucleotides were designed based on an available CBPV reference genome (Genbank EU122229 for RNA1, and Genbank EU122230 for RNA2)^6^.

Since the identities of 5′ and 3′ terminal sequences are essential for replication, we sequenced the 5′- and 3′-ends of the wtCBPV-Aut17 genome segments using a rapid amplification of cDNA end 5′-end techniques (RACE-PCR). The 5′-ends of both RNAs were determined by the addition of a homopolymeric tail to the 3′-end of a cDNA. Briefly, a first strand cDNA was synthesized using the gene specific primers CPV4 for RNA1 and CPV20 for RNA2 together with the HiScript II 1^st^ strand cDNA synthesis kit (Vazyme Biotech Co. Ltd., China). A poly-A tail was added using terminal deoxytransferase (TdT; NEB, Ipswich, USA) and dATP. Then, a first Taq DNA polymerase reaction (NEB) was started with an oligonucleotide containing an oligo dT-sequence fused to a 5′-terminal adapter sequence (CPV14) together with a gene specific primer (CPV5 for RNA1, and CPV21 for RNA2). A nested Taq PCR reaction was performed applying an adapter oligonucleotide (CPV15) and gene specific primers (CPV6 for RNA1, and CPV22 for RNA2). The PCR products were purified and cloned into a T-vector. The plasmids were purified with a plasmid preparation mini kit (Favorgen, Biotech Corp., Ping-Tung, Taiwan) and sequenced with standard oligonucleotides (M13 and M13rev).

For the determination of the 3′-end of both CBPV RNAs a DNA oligonucleotide (5′-terminal phosphorylated and 3′-terminal amino-blocked; CPV13) was ligated to the 3′-end of the viral RNAs using T4 RNA Ligase (NEB). The nucleic acids were precipitated and transcribed using CPV15, which hybridizes with CPV13. The resulting cDNA was amplified in a Taq polymerase reaction using an oligonucleotide containing the complementary adapter sequence (CPV15) together with gene specific primers (CPV10 for RNA1, and CPV30 for RNA2). A nested PCR was performed using the primer CPV12 and additional gene specific primers (CPV11 for RNA 1, and CPV29 for RNA 2). The resulting PCR products were purified, cloned into a T-vector, and sequenced as described above.

### Full-length CBPV cDNA clones

The two CBPV genome segments were cloned from single RNA molecules to exclude possible assembly errors. Therefore, genomic cDNAs of both RNA molecules were transcribed using gene specific oligonucleotides with 5′-adapter sequences (CPV7 for RNA1 and CPV23 for RNA2). Full-length PCR amplicons were generated using primers corresponding to the correct 5′- and 3′-ends (CPV8 and CPV9 for RNA1 as well as CPV24 and CPV25 for RNA2). The PCR products were purified, cloned into a pBR322 vector backbone by a DNA assembly reaction (NEBuilder, NEB), and transformed in *E. coli* (strain HB101). The complete sequences were verified by Sanger sequencing (Eurofins Genomics). Phylogenetic analysis of CBPV AUT17-CBP1 and AUT17-CBP2 and the construction of the phylogenetic tree was done with the CLC Sequence Viewer Software, Version 8.0 and available CBPV sequences from Genbank.

### RNA *in vitro* synthesis and virus rescue

Using bacterial DNA plasmids and DNA-dependent RNA polymerases, we were able to synthesize the infectious RNA molecules of CBPV. Therefore, 2 µg plasmid DNA were linearized with the restriction enzymes Sca I (AUT17-CBP1) or Sac II (AUT17-CBP2), respectively. The linearized plasmid DNA was purified and transcribed into infectious RNA using SP6-polymerase (NEB), NTPs, and the m7G(5′)ppp(5′)G RNA cap structure analogue. The volume of 0.5 µl *in vitro* synthesized RNAs from both constructs were mixed (about 1 µg total RNA) and injected into the thorax of apparently healthy-looking white-eye to blue-eye honeybee pupae (day 13 to 15 of development) using Hamilton syringes (Model 702). The pupae were incubated at 35 °C for 3 to 7 days and stored for further analysis at −80 °C.

### Marker identification

A molecular marker was required in order to clearly differentiate rCBPV from the parental wtCBPV. A diagnostic Bgl II restriction enzyme recognition site was introduced in the cDNA copy of RNA2 in plasmid AUT17-CBP2 at nt position 1649 using PCR mutagenesis (CPV35 and CPV36). This modification of the nucleotide sequence preserved the encoded amino acid sequence and served as a genetic marker within the synthetic RNA and progeny genomes. With the help of the primers CPV33 and CPV34, a 546 nt fragment was amplified flanking the Bgl II site. After purification of the PCR product, 50 ng of the DNA were digested with Bgl II and subjected to gel electrophoresis to identify the cleavage products.

### Ethics statement

The experiments were carried out with the consent of the Ethics Committee of the University of Veterinary Medicine Vienna. No animal use protocol was required by the Veterinary Office of the district of Vienna or the Federal Veterinary Office of Austria to perform this research on honey bees. No endangered or protected species were involved in the study. Privately owned land was used and accessed only after permission from the landowner and sampling was carried out only with the consent of the owner of the honey bees.

## Results

### Isolation of CBPV-Aut17

The CBPV positive sample from a colony showing typical signs of infection was used to isolate the infectious agent. Ten healthy bee pupae were infected with 1 µl of the homogenized bee lysate from the field sample containing 1.1 × 10^7^ GE CBPV. After three days of infection, the pupae were harvested and an average CBPV load of 2.7 × 10^11^ GE/bee was found. This isolated stock of wtCBPV-Aut17 was used for the sequence determination and subsequent experiments.

### Sequences of CBPV-Aut17 RNAs (AUT17-CBP1 and AUT17-CBP2)

**Figure 1:**
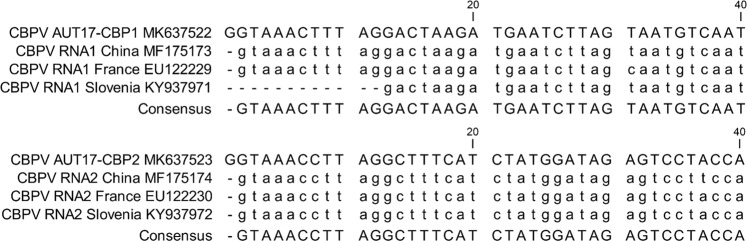
Sequence comparison of the 5′-end of RNA1 and RNA2 of CBPV-Aut17 with other genomic CBPV sequences. GenBank accession numbers are provided in the figure. Note that the complete 5′-end of RNA1 and RNA2 of the replicative molecular clone of CBPV-Aut17 shows an additional nucleotide at the very end compared to all published CBPV sequences.

**Figure 2:**
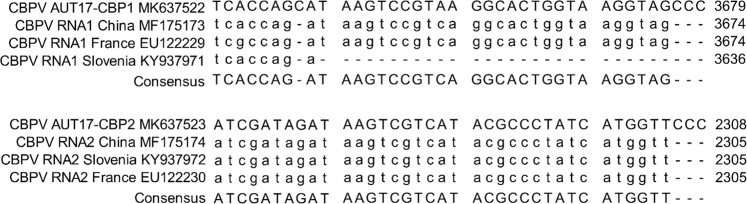
Sequence comparison of the 3′-end of RNA1 and RNA2 of CBPV-Aut17 with other genomic CBPV sequences from GenBank. GenBank accession numbers are provided in the figure. Note that the 3′ ends of both RNAs of the replicative molecular clone of CBPV-Aut17 include three additional cytosine residues compared to all other published CBPV sequences.

The RNAs were directly sequenced from RT-PCR fragments to obtain the consensus sequence using a traditional Sanger sequencing approach. For the establishment of a reverse genetics system, the PCR sequence data were verified by high-quality plasmid DNA sequencing after subsequent cloning of the PCR fragments in T-vectors. 5′- and 3′-RACE-PCRs were performed to uniquely identify the ends of the segmented genome and to review existing data. Our sequences of RNA1 and RNA2 revealed an additional guanine-residue at the 5′-end compared to published sequences from the databases. The remainder of the 5′-UTR was highly conserved between all available genomic CBPV sequences (Fig. 1). Similarly, the sequences of the 3′-ends of both RNAs contained three additional cytosine residues compared to the available genomic CBPV sequences (Fig. 2). The complete sequences of CBPV RNA1 and RNA2 have been deposited in GenBank (MK637522 for RNA1, and MK637523 for RNA2).

**Figure 3:**
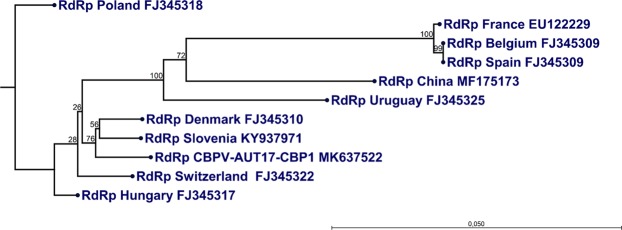
Phylogenetic analysis of the RdRp gene of CBPV-Aut17. The phylogenetic tree was constructed using the neighbor-joining method with an alignment of the conserved RdRp gene (1947 bp) of different published full-length CBPV sequences. Bootstrap values are indicated on each node as a result of 1000 replicates calculated. The phylogenetic analysis confirms the close relationship of CBPV-AUT17 to a Slovenian and a Danish isolate. GenBank accession numbers are provided.

A BLAST search showed a high similarity (between 91.73 and 98.07% for RNA1 and between 91.37 and 97.61% for RNA2) of the published genomic CBPV sequences with the sequences of our novel isolate CBPV-Aut17. Our Austrian isolate is most closely related (98.07% for RNA1 and 97.61% for RNA2, respectively) to a Slovenian CBPV strain (GenBank accession numbers KY937971 and KY937972)^27^, whose genomic sequence was decoded by Next Generation Sequencing (NGS) in 2017. We performed a phylogenetic analysis based on the ORF3 region on RNA1, which encodes for the RNA-dependent RNA Polymerase (RdRp). The resulting phylogenetic tree showed that the strain CBPV-Aut17 forms a group with the Slovenian CBPV strain from 2017 mentioned above and a Danish CBPV strain from 2007 (Fig. 3).

### ORF identification within the genome of CBPV-Aut17

The first ORF within RNA1 of CBPV-Aut17 spans from nucleotide (nt) position 20 to 2578 yielding a hypothetical (poly-)protein consisting of 853 amino acids with a predicted molecular weight of 95.1 kDa. ORF2 within RNA1 of CBPV-Aut17 starts at nt position 39 and stops at nt position 890. The hypothetical product of this coding region has 284 amino acids with a predicted molecular weight of 30.9 kDa. ORF3 of RNA1 encodes the viral polymerase. This coding region corresponds to nt position 1644 to 3590 in the Austrian isolate yielding a hypothetical protein with 649 amino acids and a predicted molecular weight of 73.8 kDa. The first ORF in RNA2 starts at nt position 23 and extends to nt position 349 encoding a hypothetical protein with 109 amino acids and a predicted molecular weight of 12.3 kDa. ORF2 of RNA2 encodes a protein termed hypothetical structural protein (hSP) consisting of 582 amino acids and spanning from nt position 297 to 2042 with a predicted molecular weight of 65 kDa. ORF 3 of RNA2 encodes a predicted structural protein, termed pSP, and spans nt position 303 to 846. The pSP gene of CBPV-Aut17 yields a translation product of 181 amino acids and a predicted molecular weight of 19.7 kDa. ORF4 of RNA2 spans nt positions 655 to 846. The hypothetical protein encoded by ORF4 has 64 amino acids and a predicted molecular weight of only 6.9 kDa. A similar ORF organization has been described for other CBPV genomes^21^.

### Establishment of a reverse genetics for CBPV

**Figure 4:**
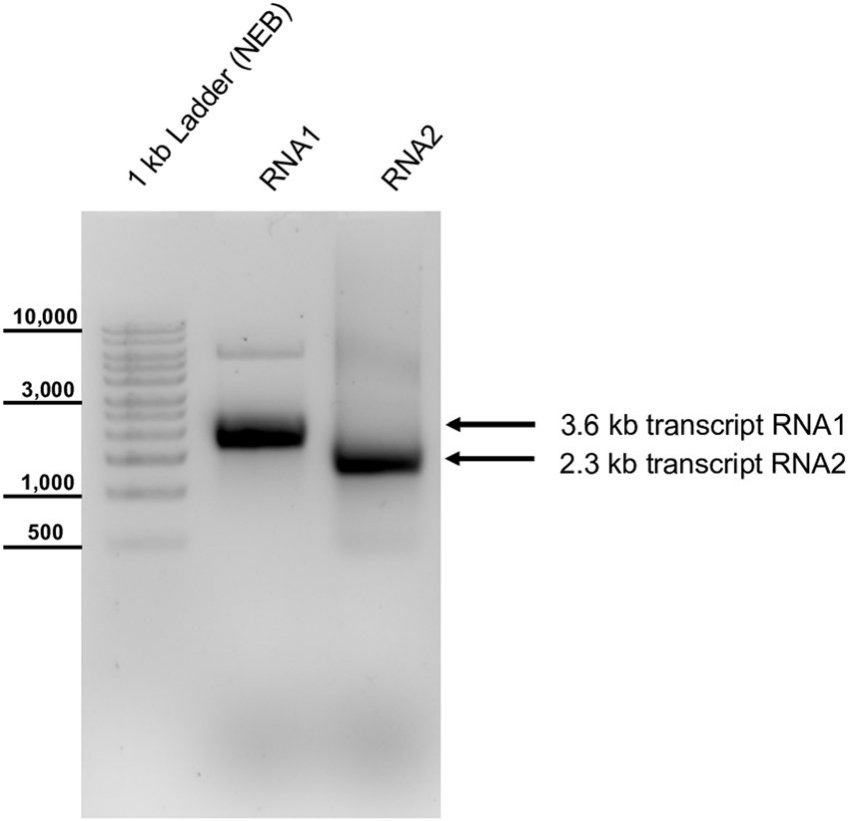
Synthetic RNA of the molecular CBPV clone. Plasmid DNAs were linearized with Sca I (RNA1) or Sac II (RNA2) and transcribed using SP6 polymerase. The synthetic RNA was injected into honey bee pupae to start the cycle of infection. Note that a DNA ladder is used for size estimation (left side).

**Figure 5:**
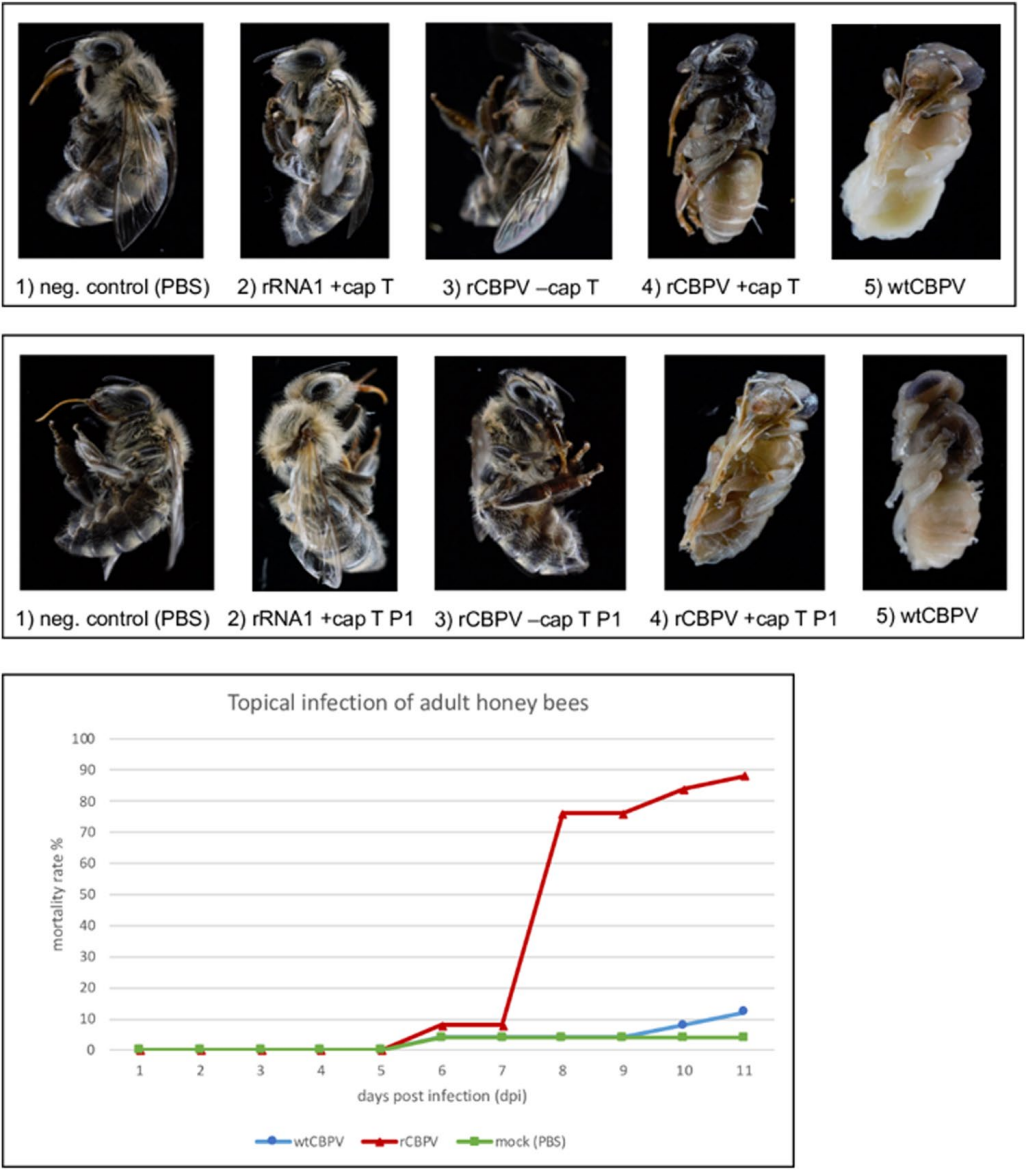
(**a**) Transfection and infection of CBPV in honey bees. Injection of pink eye honey bee pupae with PBS (1) and transfection solely with recombinant RNA1 (2) or uncapped recombinant RNA1 and RNA2 (3) resulted in the emergence of healthy-looking adult honey bees at day 21 of development. Transfection of capped recombinant RNA1 and RNA2 (4) and wild type virus suspension (5) resulted in pupal death during the incubation period. **(b)** First passage of CBPV in honey bee pupae (P1). Injection of the negative control with PBS (6), passaging of honey bee lysates from animals transfected with solely recombinant RNA1 (7), and uncapped recombinant CBPV RNA1 and RNA2 (8) resulted in the emergence of apparently healthy-looking imago at day 21 of development. Passaging of virus progeny from animals transfected with capped recombinant CBPV RNAs (9) and wild type CBPV virus suspensions (10) resulted in pupal death during the incubation period. **(c)** Topical infection of adult honey bees. Caged honey bees were topically infected with rCBPV or wtCBPV or mock infected using PBS. During the incubation period of 10 days, rCBPV infected bees showed a significantly higher mortality rate compared to wtCBPV infected bees or the negative control. Clinical signs of infection were exclusively observed in the rCBPV group starting at day 7 post infection (see Additional Video File 3).

After full-length RT-PCR, DNA amplicons of RNA1 and RNA2 of CBPV-Aut17 were inserted in a bacterial plasmid backbone (pBR322) containing all necessary elements for plasmid replication and *in vitro* RNA transcription via SP6 RNA polymerase. The integrated CBPV cDNA sequences were analyzed by Sanger sequencing. We found no base exchanges between our full-length clones of CBPV-Aut17 and the CBPV genome fragment sequences obtained from subgenomic amplicons integrated in T-vectors. This might indicate that the consensus sequence of CBPV is quite stable or that our virus strain reached a nearly clonal homogeneity in the virus isolation procedure. After linearization of the plasmid DNA with Sca I (plasmid AUT17-CBP1) and Sac II (plasmid AUT17-CBP2), we transcribed the cDNA into synthetic RNA with or without the m7G(5′)ppp(5′)G cap analogue (Fig. 4). Individual groups of 14 days old worker bee pupae were inoculated with either capped or uncapped synthetic RNAs, wild type virus suspension or PBS by an injection in the thorax. All inoculated pupae showed melanization at the injection site indicating injury and successful penetration of the pupal exoskeleton. The negative controls (injected with PBS), bees transfected with un-capped RNAs, and bees solely transfected with RNA1 or RNA2 developed into healthy imago, which emerged at day 20 to 22. In contrast, all wild type (wtCBPV) injected and recombinant viral RNA (rCBPV) transfected bees died during their pupal development. Example photographs of the results of the virus passage and infection experiments are shown (Fig. 5a,b). No difference was observed between the rCBPV and wtCBPV with respect to pathogenicity in bee pupae. Viral titers of all animals were assessed as genome equivalents (GE/bee) using RT-qPCR (Table 2).

**Table 2:** Evaluation of qRT-PCR titers of honey bee pupae after transfection and infection with CBPV*.

	GE transfected	GE/bee T	GE/bee P1	GE/bee P2
wtCBPV	—	—	2.73 × 10^11^	1.86 × 10^12^
rCBPV capped	9.80 × 10^10^ RNA1 8.34 × 10^10^ RNA2	8.53 × 10^12^	4.14 × 10^12^	1.34 × 10^13^
rCBPV uncapped	7.80 × 10^10^ RNA1 6.64 × 10^10^ RNA2	2.25 × 10^8^	n.d.	n.d.
RNA1 capped	1.96 × 10^11^	3.04 × 10^8^	n.d.	n.d.
mock	n.d.	n.d.	n.d.	n.d.

The replication and spread of rCBPV suggests the successful establishment of a CBPV reverse genetics system, with average viral loads of 8.5 × 10^12^ GE/bee in the pupae transfected with both capped rCBPV RNA molecules. We diluted the sterile filtered lysate of a transfected pupae 1,000 fold resulting in a dose of 1.0 × 10^8^ GE/µl. The diluted bee lysate (1 µl) was injected into freshly prepared pink eye bee pupae (passage 1; P1). An average viral load of 4.1 × 10^12^ GE/bee was measured after an incubation period of seven days in this first passage of rCBPV. We performed a second virus passage (P2) to study viral replication in more detail, again diluting the lysate of an infected pupa from P1 1,000 fold resulting in a dose of 1.3 × 10^7^ GE/µl. The P2 infection experiment yielded average titers of 1.3 × 10^13^ GE/bee indicating similar virus growth in different passages. The wtCBPV was passaged in parallel using 1 µl of bee lysate as described above. The viral titers of capped rCBPV were comparable to viral titers of wtCBPV (2.7 × 10^11^ GE/bee) in the passage experiment. A mortality rate of 100% in bee pupae was observed for capped rCBPV and wtCBPV confirming the high pathogenicity of the isolate CBPV-Aut17 (Fig. 5b).

No nucleic acids of CBPV RNA1, which includes the target sequence for the RT-qPCR assay, were detected in the negative controls and after transfection of RNA2 alone. The transfection of both uncapped RNA molecules yielded an average dose of 2.3 × 10^8^ GE/bee, most likely resulting from residual DNA template molecules used in the transcription reaction. Similar GE-loads were measured after transfection of capped RNA1 alone (3.0 × 10^8^ GE/bee). We further assessed progeny virus production after transfection of these synthetic RNAs by virus passaging. For this purpose, we injected 1 µl of the undiluted sterile filtrated lysates of pupae transfected with uncapped RNAs or solely transfected with capped RNA1 in freshly prepared pupae. No nucleic acids of CBPV RNA1 were detectable in P1 of these transfections. Additionally, the absence of other common honeybee viruses (SBV, ABPV, and DWV) was confirmed by RT-PCR.

To assess the replication kinetics of rCBPV-Aut17 and wtCBPV-Aut17 in adult honey bees, three groups of 25 newly emerged bees were caged as described above (day 0). At day 1 of the experiment, we infected the bees by topical application of 1,0 × 10^9^ GE of virus suspension obtained from the first passage of rCBPV (P1) and wtCBPV. After an incubation period of ten days the bees were harvested and the viral titer was assessed, reaching an average value of 1.63 × 10^12^ GE/bee for rCBPV and 5.83 × 10^11^ GE/bee for wtCBPV-Aut17 (Table 3). The experiment confirmed the successful replication of the recombinant virus in adult honey bees. During the experiment, rCBPV infected bees showed a significantly higher mortality rate compared to the wtCBPV infected bees and the negative control (Fig. 5c). After 7 days post infection, the remaining rCBPV infected bees started showing typical signs of infection, such as discoloration and trembling (Additional Video File 3). Obvious differences in the mortality rate of bees infected with rCBPV and wtCBPVV correlated with the higher virus titer of rCBPV infected animals.

**Table 3:** Evaluation of qRT-PCR results of adult honey bees after topical infection with CBPV*.

	Average	Median	Minimum	Maximum
wtCBPV	5.83 × 10^11^	1.85 × 10^7^	2.37 × 10^6^	4.05 × 10^12^
rCBPV	1.63 × 10^12^	1.49 × 10^12^	3.07 × 10^10^	6.70 × 10^12^
mock (PBS)	n.d.	n.d.	n.d.	n.d.

*Abbreviations: n.d. = not detected, GE = genome equivalents, wt = wild-type, r = recombinant, T = transfection, P = passage.

### Genetic marker identification

**Figure 6:**
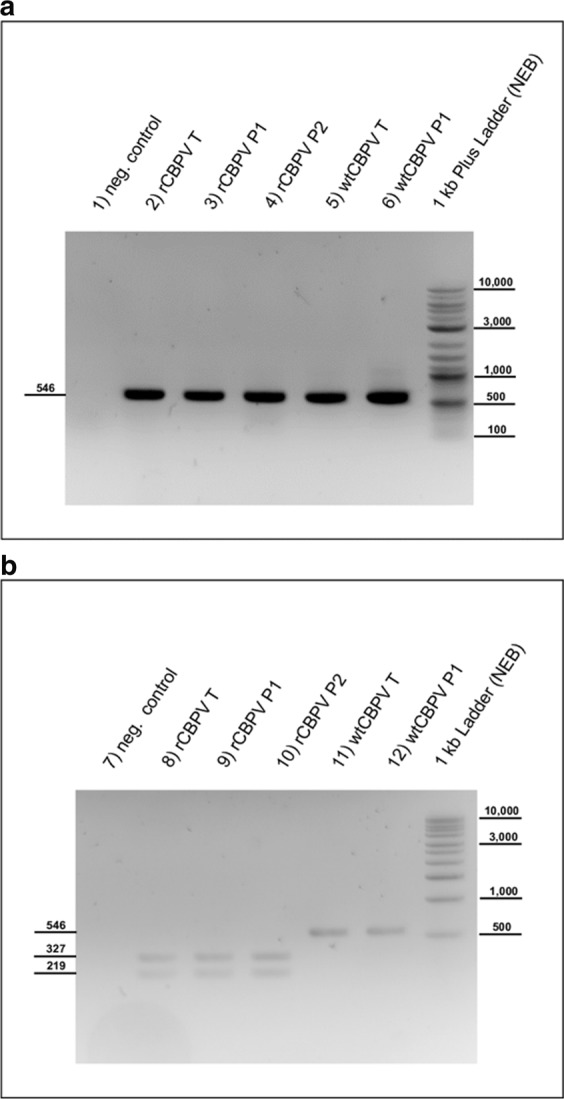
Identification of the Bgl II restriction site in rCBPV RNA2. **(a**) A RT-PCR fragment flanking a Bgl II site, which was introduced in the rCBPV sequence as a genetic marker, was performed using the primers CPV33 and CPV34. Pupae transfected with recombinant CBPV (lane a2), infected with passaged rCBPV (a3, a4) or infected with wild type CBPV (lane a5 and a6) show an RT-PCR product of the expected size (546 bp). PBS injected pupae serve as negative controls with no visible amplicon (lane a1 and b1). **(b)** Digestion of the RT-PCR products with Bgl II yielded two distinct bands of 327 and 219 bp in the case of recombinant CBPV (lane b8-b10), compared to wtCBPV, where no cleaved PCR product occurred (lane b11 and b12).

We introduced a novel Bgl II restriction endonuclease site in the cDNA of RNA2 serving as a genetic marker. This site allowed to distinguish between wtCBPV and rCBPV. After Bgl II digestion of RT-PCR products, two distinct bands (327 and 219 nts) became visible in all rCBPV infected animals and no undigested DNA remained. This experiment confirmed the stability of our genetic marker, the absence of wild type virus pre-infections, and the successful rescue of rCBPV (Fig. 6).

## Discussion

In this study, we isolated CBPV from a typical field case in Austria and generated a functional molecular clone that induced chronic bee paralysis as a basis for reverse genetics. Traditional genetic analysis of viruses (forward genetics) focuses on a specific phenotype and tries to correlate this phenotype with the existence of a specific genotype by means of genetic analysis. Reverse genetics depends on molecular cloning and recombinant DNA technology and takes an opposite approach. Reverse genetics therefore begins with a cloned DNA molecule of a defined sequence, which is used to generate mutant genes that eventually produce a mutant phenotype. This makes it easier to decipher the functions of genes and to identify viral virulence factors. At the same time, reverse genetics avoids the experimental uncertainties arising from the heterogeneity within RNA virus species clouds. Reverse genetics systems have provided powerful tools to study all aspects of the virus biology and virus-host interactions^28^.

As we have recently shown for DWV, one important key factor for generating a molecular clone of CBPV was to obtain the complete sequence of the genome segments applying established 5′- and 3′- RACE PCR methods. Our data neither support the existence of a strong secondary structure as found in internal ribosomal entry sites (IRES) nor a protein at the 3′-ends of the virus genome, as hypothesized by other studies, as the ligation of an adapter primer on the RNA and the subsequent amplification of the 3′-end was possible without hindrances^6^. Our reverse genetics system for CBPV consisted of two plasmids that allowed the separate transcription of both full-length genome segments. Transfection of both synthetic RNAs with synthetic 5′- cap structures resulted in viral replication, production of infectious virus progeny, and 100% mortality of the transfected honey bee pupae. In our experiment, solely the synthetic transcripts including the 7-methyl-guanosine cap structure analogue were replicative after transfection and produced progeny virus. Hence, we directly demonstrate the importance of the 5′ -cap structure for CBPV replication confirming previous assumptions^6^.

By introducing a genetic marker, we ensured the differentiation of our recombinant progeny virus from wild-type CBPV. The diagnostic restriction enzyme sequence was stably retained within RNA segment 1 over three passages. The recombinant CBPV replicated as efficiently as its parental isolate, reaching average viral titers of 8 × 10^12^ GE/bee after transfection. Interestingly, rCBPV and wtCBPV both produced high viral titers in honey bee pupae. These experimental results are not only in sharp contrast to an earlier study that reported very low viral titers in naturally infected honey bee pupae and larvae^12^, but are also in conflict with the hypothesis that CBPV is primarily a pathogen of adult honey bees. Future studies will test the infection of pupae and larvae under more natural conditions to study the biological significance of larval and pupal infections. Laboratory systems are going to be used, in which infected adult bees feed the brood in a controlled cage setup, to investigate CBPV transmission and pathogenicity in the bee brood. In addition, the infection of pupae by Varroa mites contaminated with CBPV will be further investigated. At this point, we cannot exclude that the observed virulence of CBPV in honey bee pupae is caused by the individual properties of the virus isolate CBPV Aut17. Therefore, the same approach using cDNAs of different CBPV strains will be performed in the future. Using topical infection, rCBPV was able to induce clinically relevant disease and high mortality in adult honey bees. As documented before, a sharp increase in mortality of the caged bees was observed after day 6 of infection and typical signs of CBPV were documented^29^. Surprisingly, neither the typical signs of the disease nor a comparable mortality rate were observed after the topical infection of adult honey bees with wtCBPV although an equal amount of genome equivalents was used. During the evaluation of these experiments, it was noticed that a significantly lower virus titer occurred in the wtCBPV bees after infection. We therefore assume that the wtCBPV stock solution had a lower effective infection dose, although it contained the same number of RNA molecules of CBPV RNA (measured by RT-qPCR in GE). Since there are no suitable cell culture systems for CBPV, we did not determine the real infection dose for this experiment (for example measured as TCID_50_), so this point cannot be conclusively clarified. For this reason, one focus of further work will be on developing methods for quantifying the infection dose of CBPV, like virus titration in honey bee pupae.

Clinically relevant outbreaks of CBPV and the infection dynamics within the colonies are still poorly understood. The virus is widespread in the field and able to persist in colonies in covert infection cycles causing no obvious signs of disease^8,9,15^. Yet we have convincing evidence that CBPV infections of honey bee pupae via injection with RNAs or viral particles have a fatal outcome, confirming earlier observations about CBPV being competent of inducing overt disease with massive losses of honey bees^11^. The extent of adult honey bee losses during the summer due to CBPV might be underestimated and it was assumed that a relevant proportion of all bee deaths among workers could be caused by CBPV, even in the covert infection scenario^8,13^. Further research is crucial to resolve the impact of CBPV on the honey bee population and to combat massive losses of worker bees during overt CBPV outbreaks. The establishment of a reverse genetics system is a first step towards elucidating the mechanisms of transmission, replication and pathogenicity of this interesting bee virus. The cDNA copy of one defined genome allows to produce a homogenous virus population and further enables a targeted manipulation of the CBPV genome *in vitro* to unravel the factors of pathogenicity of this virus.

Koch’s postulates were formulated in 1884 based on Henle’s older concepts about infectious agents. The postulates are four criteria that must be met to establish a causal relationship between a microbe and a disease. In many approaches, attempts have been made to adapt the postulates to viral agents and the progress of modern infection research^30–33^, although the main features have always been retained: The pathogen must be detected in organisms suffering from the disease, but not regularly in healthy organisms. A pure culture of the pathogen should cause disease if it is properly inoculated into a healthy organism and the multiplication of the pathogen in the diseased host must be demonstrated showing that the pathogen is indeed identical. In the case of CBPV, no defined pure cultures have been established yet due to a lack of cell culture systems. In a strong approach to Koch’s postulates, virus particles from CBPV bee cultures have been purified, the two RNAs extracted and inoculated into experimental animals^20^. Although this approach provided very strong evidence of a link between CBPV and disease symptoms, the possibility remained that contamination could falsify the results. The infectivity of fully synthetic RNAs, on the other hand, can be used as irrefutable evidence of the causal relationship between CBPV and clinical signs. Our CBPV clone satisfies Koch’s postulates and will be a valuable tool for the in-depth analysis of CBPV pathogenesis.

## Supplementary information

Additional Video File 3

Additional Video File 2

Additional Video File 1

## Data Availability

All data generated or analyzed during this study are included in this published article (and its Supplementary Information files).
